# The importance of extranodal extension in penile cancer: a meta-analysis

**DOI:** 10.1186/s12885-015-1834-4

**Published:** 2015-10-28

**Authors:** Zhi-Ling Zhang, Chun-Ping Yu, Zhuo-Wei Liu, Liliya Velet, Yong-Hong Li, Li-Juan Jiang, Fang-Jian Zhou

**Affiliations:** 1 Department of Urology, Sun Yat-sen University Cancer Center, Guangzhou, Guangdong 510060 P. R. China; 2 State Key Laboratory of Oncology in South China, Collaborative Innovation Center for Cancer Medicine, Guangzhou, Guangdong 510060 P. R. China; 3 Department of Nephrology, Guangdong General Hospital, Guangdong Academy of Medical Sciences, Guangzhou, Guangdong 510080 P. R. China; 4 Northeast Ohio Medical University, Rootstown, OH 44272 USA

**Keywords:** Extranodal extension, Penile cancer, Prognosis, Meta-analysis, Pelvic lymph node metastasis

## Abstract

**Background:**

The role of extranodal extension (ENE) in penile cancer is controversial and has not been well studied. The aim of this study was to investigate the importance of ENE in predicting prognosis and presence of pelvic lymph node metastasis (PLNM) in penile cancer patients.

**Methods:**

We searched related studies in Medline, Embase, Cochrane Library, and Scopus database. Hazard ratio (HR) and odds ratio (OR) were directly extracted or indirectly estimated from the included studies.

**Results:**

A total of ten studies with 1,142 patients were included in this meta-analysis. Patients with ENE showed a worse cancer-specific survival (CSS) (HR = 1.90, 95 % confidence interval [CI] = 1.35–2.67, *P* = 0.0002) and overall survival (HR = 4.04, 95 % CI = 1.02–16.1, *P* = 0.05) than those without ENE. Further subgroup analysis revealed that the predictive value of ENE for CSS in penile cancer patients was significant regardless of the study’s country of origin, but not in the subgroup with shorter follow-up time (<36 months, *P* = 0.38). Patients with ENE also showed a higher incidence of presenting with PLNM (OR = 4.95, 95 % CI = 2.58–9.49, *P* < 0.001). A stratified analysis demonstrated that the predictive role of ENE for PLNM was only detected in studies with a larger sample size (> 100 cases). No significant publication bias was observed, as suggested by Begg’s and Egger’s tests.

**Conclusions:**

ENE is associated with worse prognosis and high risk of PLNM in penile cancer patients. Due to the limited number of studies included in this meta-analysis, a large-scale, well-designed study will be required to verify our results.

**Electronic supplementary material:**

The online version of this article (doi:10.1186/s12885-015-1834-4) contains supplementary material, which is available to authorized users.

## Background

Penile cancer is an uncommon malignancy, with an incidence of less than 1/100,000 in Western countries [1, 2]. The prevalence of penile cancer is higher in developing regions, yet the overall tendency is showing a decrease in morbidity [3–5]. It is imperative to identify prognostic factors of this disease, however due to its rarity this task is difficult. Generally, the most widely accepted prognostic factor of penile cancer is the status of the regional lymph nodes [6–8]. The 7th American Joint Committee on Cancer (AJCC) cancer staging manual [9] categorizes patients with a single positive inguinal lymph node as pN1, and multiple as pN2. Penile cancer with inguinal lymph node metastasis (LNM) shows a poorer prognosis than those without inguinal LNM. In addition, the prognosis of patients with pelvic lymph nodes metastasis (PLNM) is even worse [10–12].

Extranodal extension (ENE) is defined as extension of tumor through the lymph node capsule into the perinodal fibrous-adipose tissue. Along with the number and location of LNM, ENE is also considered a negative prognostic factor in penile cancer patients. In the latest AJCC TNM staging [9], both ENE and PLNM are staged as pN3, suggesting that ENE is an extremely terrible pathologic finding. However, the role of ENE in penile cancer is still controversial. Certain studies have indicated that penile cancer patients with ENE had a lower 5-year survival rate compared with those without ENE [13–16]. Several other reports, however, find that ENE is not an independent prognostic factor [17, 18]. As for PLNM, the European Association of Urology (EAU) guideline recommends considering ENE in inguinal lymph nodes as a risk factor for PLNM [19], even though some studies have failed to find a significant predictive role of ENE for PLNM [16, 20, 21]. The above-mentioned contradictory results contribute to the uncertainty of the role of ENE in penile cancer.

These controversial results regarding ENE may be attributed to the rarity of penile cancer. Both the number of patients and statistical power in each individual study is limited. In the current study, we performed a meta-analysis that pooled together all the related studies focusing on the role of ENE in penile cancer. We aimed to better illuminate the importance of ENE in predicting survival and the risk of PLNM in penile cancer patients.

## Methods

This meta-analysis was preformed following the Preferred Reporting Items for Systematic Reviews and Meta-analyses (PRISMA) criteria [22].

### Literature search strategy

We performed a search in the Medline, Embase, Cochrane Library and Scopus databases to identify relevant studies for this meta-analysis. Our search duration lasted until November 18, 2014. We utilized the following terms and combinations for searching: “penile cancer or carcinoma of penis or penis cancer or penile neoplasms” and “extranodal or extra capsular or capsular penentration or perinodal”. Furthermore, references of retrieved articles and reviews were manually screened for additional studies.

### Inclusion and exclusion criteria

Studies were considered eligible if they met both of the following criteria: (1) published in English and (2) reported the association between ENE and prognosis and/or the risk of PLNM of penile cancer. Studies were excluded based on the following criteria: (1) letters, case reports, reviews, and conference abstracts; (2) studies which did not provide sufficient information to estimate hazard ratio (HR) or odds ratio (OR) and 95 % confidence interval (CI); and (3) studies with duplicated data or a repeated analysis. When the same group reported duplicated data in papers, we only included the most informative one.

### Data extraction and quality assessment

We extracted the useful data from eligible studies by using a standard information collection survey including the following items: first author’s name, publication year, recruitment period, country of origin, follow-up time, number of patients, number of patients with ENE, and HR/OR with 95 % CI. The quality of the included studies was assessed by the Newcastle-Ottawa Scale (NOS) [23]. Studies with seven or more stars were defined as high quality studies. Two authors (Zhi-Ling Zhang and Chun-Ping Yu) independently reviewed the above data, and all disparities were resolved by discussions between the authors.

### Statistical analysis

In order to analyze the impact of ENE on the survival of penile cancer patients, we synthesized survival data from the included studies using the reported HR and its 95 % CI. When the survival data was not directly reported, we used a mathematical estimation to calculate the necessary data with the methods reported by Tierney et al. [24]. By convention, an observed HR > 1 implied worse survival for patients with ENE. The association between ENE and PLNM was evaluated by OR. All numbers needed for calculating OR and their 95 % CIs were directly extracted from the multivariable logistic regression or univariate analysis. If a study provided both, the results of multivariable and univariate analysis, we used the former. We used Higgins *I*^*2*^statistic to quantify the heterogeneity of the pooled results. If it was *I*^2^ < 50 %, indicating the absence of heterogeneity, then a fixed-effects model was used to estimate the pooled HRs/ORs. Otherwise, the random-effects model was used. Subgroup analysis was used to detect the potential heterogeneity among studies. Begg’s funnel plot and Egger’s linear regression test were conducted to examine publication bias in the literature [25, 26]. A sensitivity analysis was conducted to confirm the robustness of the pooled results, during which data from each individual study was sequentially removed. All *P* values were two-tailed and the *P* values of <0.05 were considered statistically significant. The statistical analysis was conducted using STATA 11 (StataCorp, College Station, Texas, USA).

## Results

### Summary of analyzed studies

**Fig. 1:**
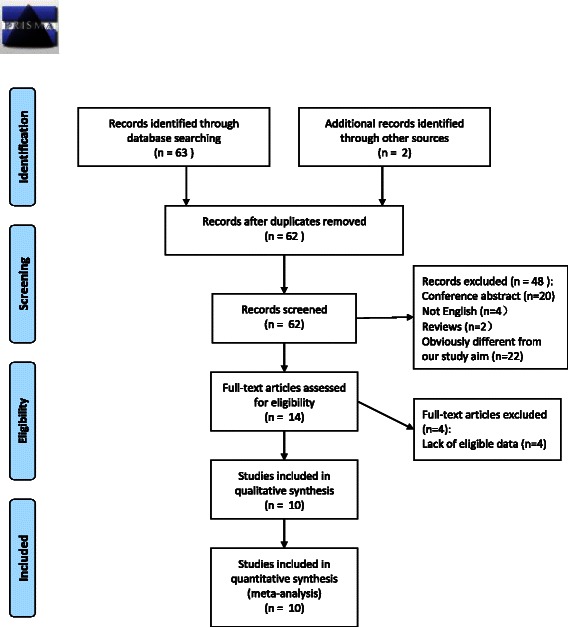
Preferred Reporting Items for Systematic Reviews and Meta-Analyses (PRISMA) Flow diagram

The present study met the PRISMA statement (Additional file 1). In total, 65 studies were identified from an initial search, and 3 studies were excluded for duplicated reporting. All the 62 studies were screened and 48 studies were excluded by screening of titles and abstracts. The remaining 14 original studies were then reviewed by careful screening of the full texts, after which 4 were eliminated due to lack of eligible data. Finally, 10 studies were chosen to be included in this meta-analysis (Fig. 1).

Table 1 summarized the main characteristics of the included studies. A total of 1,142 patients were included in this meta-analysis, ranging from 33 to 300 in each individual study. Of the 10 eligible studies, 4 were carried out in Asian [14, 16, 21, 27], 4 in European [13, 18, 20, 28], and 2 in North American [15, 17] countries; 6 studies reported the association between ENE and prognosis [13–15, 17, 18, 27], 3 studies investigated the relationship between ENE and PLNM [20, 21, 28], and 1 study focused on both [16]. In the 7 studies that provided prognostic information [13–18, 27], 4 used cancer specific survival (CSS) [13, 15, 17, 18], 2 used overall survival (OS) [14, 16], and only 1 used recurrence-free survival [27]; 5 listed HR and its 95 % CI in multivariable Cox regression [13, 14, 17, 18], and 2 provided survival curves for calculating them [15, 27]. ORs and 95 % CIs were directly extracted from 3 studies [16, 20, 28] and calculated indirectly in 1 study [21]. The quality of the included studies was evaluated, revealing that 9 (90 %) were high quality and 1 (10 %) was moderate.

**Table 1:** Main characteristics of the studies included in this meta-analysis

First author	Year	Country	Recruitment period	Study design	Age (median)	Follow-up (mean/median)	No. pts	No. ENE Pts (%)	No. PLND Pts (%)	No. PLNM Pts (%)	Outcomes measured	NOS score
Djajadiningrat [13]	2014	Netherlands	1956–2012	retrospective	64	65 m	300	134(45)	NA	58(19)	CSS: Multivariable HR reported	8
Lughczznni (a) [18]	2013	Italy	2000–2012	retrospective	61	26 m	81	40(49)	56(69)	NA	CSS: Multivariable HR reported	8
Pandey [14]	2006	India	1987–1998	retrospective	45	NA	102	54(53)	NA	21(22)	OS: Multivariable HR reported	8
Svatek [17]	2009	USA	1979–2007	retrospective	61	24 m	45	11(24)	33(73)	NA	CSS: Multivariable HR reported	8
Sun [15]	2014	Canada	1994–2010	retrospective	65	43 m	155	71(46)	NA	31(7.1)	CSS: Curve estimated HR	7
Zhu(a) [27]	2011	China	1990–2008	retrospective	48	53 m	60	16(27)	29(48)	8(13)	RFS: Curve estimated HR	7
Liu [16]	2013	China	1998–2011	retrospective	51	42 m	76	29(38)	42(55)	33(43)	OS: Multivariable HR reported	6
											PLNM: Multivariable OR reported	
Zhu(b) [21]	2008	China	1990–200	retrospective	NA	38 m	33^a^	5(15)	16(48)	12(36)	PLNM:univariateOR calculated	7
Lont [20]	2007	Netherlands	1956–2011	retrospective	63	85 m	102	NA	53(52)	25(25)	PLNM: Multivariable OR reported	8
Lughczznni (b) [28]	2014	Italy	1985–2012	retrospective	63	51 m	188^b^	98(52)	160(85)	51(27)	PLNM: Multivariable OR reported	8

*NA* not available, *ENE* extranodal extension, *CSS* cancer specific survival, *OS* overall survival, *RFS* recurrence free survival, *PLND* pelvic lymph node dissection, *PLNM* pelvic lymph node metastasis, *OR* odds ratio, *HR* hazard ratio, *NOS* Newcastle-Ottawa Scale

^a^30 patients and 33 groin basins;^b^142 patients and 188 groin basins

### Association between ENE and survival in penile cancer patients

**Fig. 2:**
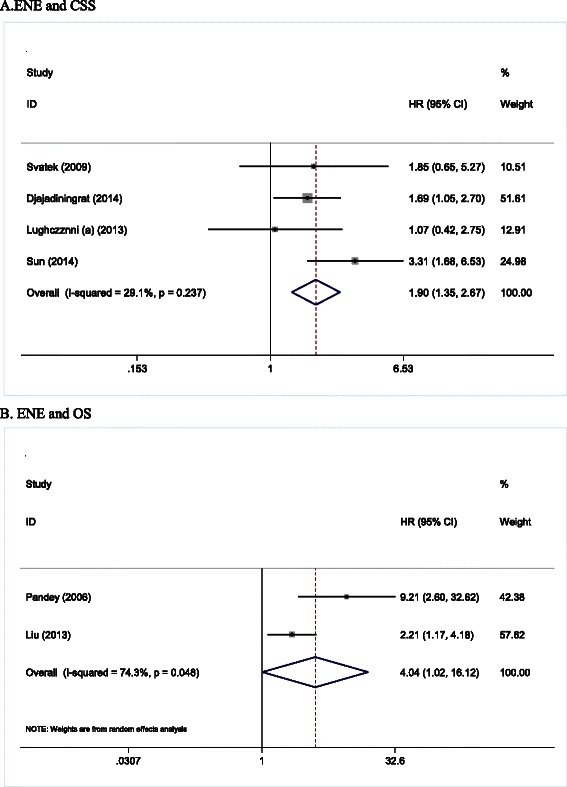
Meta-analysis of the pooled HRs of CSS (**a**) and OS (**b**) for penile cancer patients with ENE. HR > 1 implied ENE was significantly associated with worse prognosis. CSS, cancer specific survival; OS, overall survival; ENE, extranodal extension; CI, confidence interval

Since there was only one study that used recurrence-free survival to evaluate the prognosis, it was excluded from the prognostic analysis [27]. The overall analysis revealed that patients with ENE had a worse CSS compared to those without ENE (HR =1.90, 95 % CI = 1.35–2.67, *P* < 0.001) (Fig. 2a). Furthermore, we performed a subgroup analysis looking at the study’s country of origin and follow-up time. We found a significant association between ENE and CSS in both European (HR = 1.54, 95 % CI: 1.01–2.36) and North American (HR = 2.79, 95 % CI: 1.58–4.92) cohorts. Then, we subdivided studies based on follow-up time. We found that the association between ENE and CSS was only present in studies with a median follow-up time longer than 36 months, but not in those with shorter follow-up time (Table 2).

**Table 2:** Stratified analysis of pooled hazard ratios for penile cancer patients with ENE

Analysis	No. of studies (No. of patients)	HR/OR (95 % CI)	*P* Value	Model	Heterogeneity	
					I^2^ (%)	P_het_
Whole group CSS	4(581)	1.90(1.35,2.67)	0.0002	Fixed	29	0.24
Subgroup1:						
Location						
European	2(381)	1.54 (1.01, 2.36)	0.05	Fixed	0 %	0.40
North American	2(200)	2.79 (1.58, 4.92)	0.0004	Fixed	0 %	0.36
Subgroup2:						
Follow-up time						
< 36 months	2(126)	1.37 (0.68, 2.76)	0.38	Fixed	0 %	0.45
≥ 36 months	2(455)	2.26 (1.18, 4.34)	0.01	Random	60 %	0.11
Whole group PLNM	4(353)	4.95 (2.58, 9.49)	<0.00001	Fixed	0	0.51
Subgroup1:						
Sample size						
≥ 100	2(244)	5.54 (2.63, 11.68)	<0.00001	Fixed	0 %	0.44
< 100	2(109)	3.43(0.90, 13.11)	0.07	Fixed	26 %	0.25
Subgroup2:						
Identification of study object						
Individual patient	2(178)	2.82 (0.99, 7.98)	0.05	Fixed	0 %	0.71
Groin basin	2(221)	7.11 (3.08, 16.38)	<0.00001	Fixed	0 %	0.56

*ENE* extranodal extension, *CSS* cancer specific survival, *PLNM* pelvic lymph node metastasis, *OR* odds ratio, *HR* hazard ratio, *CI* confidence interval

For OS, patients with ENE showed a lower OS rate compared with those without ENE (HR = 4.04, 95 % CI: 1.02–16.1, *P* = 0.05) (Fig. 2b). Only two studies were included in this analysis, therefore a stratified analysis was not performed.

### Association between ENE and PLNM in penile cancer patients

**Fig. 3:**
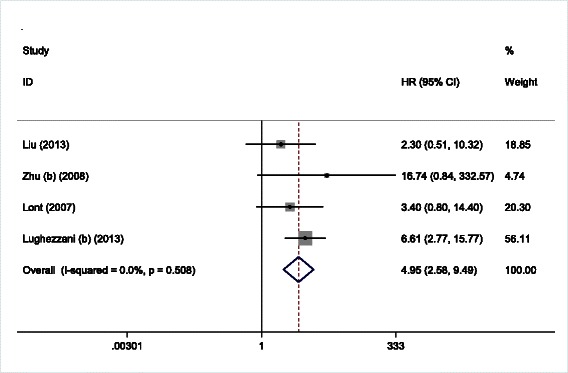
Forest plots of the OR for the association of ENE with PLNM of penile cancer patients. OR > 1 indicates that ENE was significantly associated with high risk of PLNM. OR, odds ratio; ENE, extranodal extension; PLNM, pelvic lymph node metastasis; CI, confidence interval

Four studies investigated the relationship between ENE in inguinal lymph nodes and the presence of PLNM. Our meta-analysis revealed that patients with ENE had a higher risk of presenting with PLNM (Fig. 3, OR = 4.95, 95 % CI: 2.58–9.49, *P* < 0.001) and significant heterogeneity was not found (*I*^2^ = 0 %, *P* = 0.508). Subgroup analysis revealed that the predictive role of ENE for PLNM was only observed in studies with a sample size larger than 100 (OR = 5.54, 95 % CI: 2.63–11.68, *P* < 0.001). We also took into consideration what each study identified as its study object. For example, some studies identified the patient as the object, whereas others used the individual groin basins of the patient as the object. Our analysis revealed that pooled HR for the “one patient as a study object” group was 2.82 (95 % CI = 0.99–7.98, *P* = 0.05), while, the “one groin basin as a study object” group was 7.11 (95 % CI = 3.08–16.38, *P* < 0.001).

### Publication bias

**Fig. 4:**
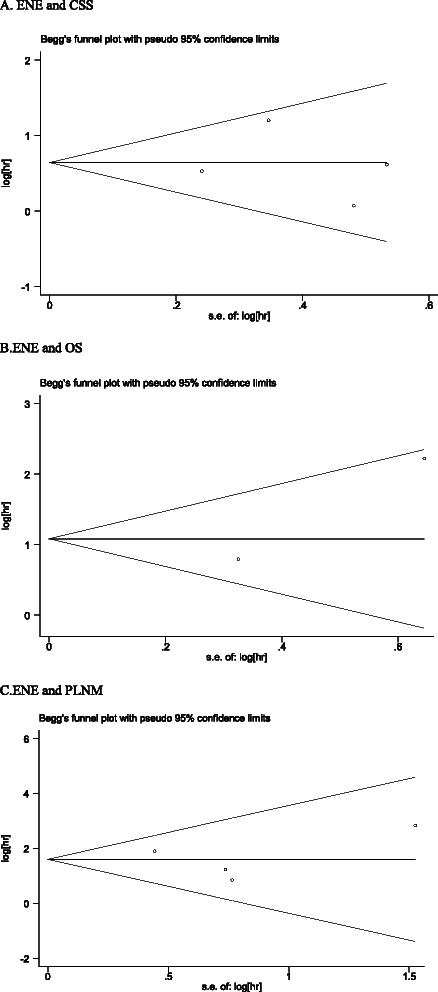
Funnel plot for all studies included in this meta-analysis. **a** and **b** funnel plot assessing ENE and cancer specific survival and overall survival, respectively; **c** funnel plot assessing ENE and pelvic lymph node metastasis in penile cancer patients. ENE, extranodal extension; SE, standard error

We used Begg’s funnel plot and Egger’s test to evaluate the publication bias of all the relevant studies. As shown in Fig. 4, the shape of the funnel plot was symmetrical for the all comparisons, revealing that there was no obvious publication bias. For the relationship between ENE and CSS, the p value of Begg’s test was 1.000 and 0.890 for Egger’s test. The p value of Begg’s test for ENE and OS was 1.000, and the p value of Begg’s test was unavailable due to the limited number of studies. Similarly, the statistical results did not show evidence of publication bias for PLNM (Begg’s test, *P* = 0.734; Egger’s test, *P* = 0.951).

### Sensitivity analysis

In order to evaluate the stability of our results, we performed a sensitivity analysis by removing one study at a time. The results of the sensitivity analysis were shown in Table 3. The corresponding pooled HR/OR did not significantly change after sequentially omitting each study, demonstrating that our results were stable and reliable.

**Table 3:** Sensitivity Analysis for CSS and PLNM

	Study omitted	HR or OR (95 % CI)	*P* Value
CSS	Djajadiningrat [13]	2.16 (1.33, 3.51)	0.002
	Lughczznni (a) [18]	2.08 (1.44, 2.99)	<0.0001
	Svatek [17]	1.91 (1.33, 2.74)	0.0004
	Sun [15]	1.58 (1.07, 2.34)	0.002
PLNM	Liu [16]	5.91 (2.87, 12.18)	<0.00001
	Zhu(b) [21]	4.66 (2.39, 9.08)	<0.00001
	Lont [20]	5.44 (2.62, 11.29)	0.01
	Lughczznni (b) [28]	3.42 (1.28, 9.13)	<0.00001

*CSS* cancer specific survival, *PLNM* pelvic lymph node metastasis, *HR* hazard ratio, *OR* odds ratio, *CI* confidence interval

## Discussion

As early as 1987, Srinivas et al. [6] reported that lymph node positive penile cancer with ENE was associated with a higher mortality than penile cancer without ENE. Subsequent studies reported a 5-year survival rate of 9 %–42 % [14, 29] in penile cancer patients with ENE, comparable to those with PLNM [10, 16, 28, 29]. Hence, in the latest AJCC cancer staging system, both ENE and PLNM were listed as pN3 stage. Several studies indicated that ENE was not only a predictor of poor survival [13–16], but also a risk factor for PLNM [18]. However, the reports on the role of ENE were not consistent, and some studies failed to find an independent predictive value of ENE in predicting prognosis or PLNM. For example, Lughezzani et al. [28] used multivariable Cox regression models to identify the independent predictive factors of CSS in penile cancer. They found that patients with ENE showed a worse CSS with a HR of 1.069 and 95 % CI: 0.416–2.750, while the difference in prognosis was not statistically significant (*P* = 0.889). Similar results were reported by Svateket al.[17]. Moreover, three studies that focused on the role of ENE in predicting PLNM failed to find a significant association [16, 20, 21]. In order to better illustrate the role of ENE in penile cancer, we performed the present meta-analysis. Our study shows advantages compared to the individual studies, which are limited by small sample size and insufficient statistical power, thereby clarifying the importance of ENE in penile cancer patients. The results of the present study reveal that penile cancer patients with ENE have worse CSS and OS, as well as higher risk of presenting with PLNM, compared to those without ENE.

Since the patient characteristics were different in each included study, we performed a stratified analysis based on the location of the study to detect the role of ENE in penile cancer. Our results showed that the predictive role of ENE in CSS was observed in both, European and North American subgroups. This may be partly explained by the same race of the two regions. Another reason for this phenomenon may be attributed to the similar treatment protocol in these two regions. We also found that the association between ENE and CSS was significant only in the studies that had a median follow-up time of no less than 36 months. It is not difficult to understand this finding of increased mortality with a prolonged follow-up time.

A subgroup analysis looking at the association between ENE and PLNM revealed that ENE was associated with PLNM in studies with a sample size larger than 100. This finding is consistent with the basic statistical principle that statistical difference may be left unrecognized in small sample studies. Our analysis also reveals an interesting finding regarding the differences in how the study object is defined. The subgroup analysis showed that studies using one groin basin as the study object had a significant predictive value of ENE for PLNM. However, the studies that utilized one patient as a study object did not obtain a statistically significant pooled OR. The pattern of regional lymph node metastasis in penile cancer is unique. Primary tumor first metastasizes to unilateral, bilateral or contralateral inguinal lymph nodes, after which metastasis can extend to the ipsilateral pelvic lymph nodes. No skip metastasis has been observed [19]. According to this observation, it is theoretically more reasonable to predict PLNM using groin basins as the study object. Our results confirm this speculation.

When investigating the relationship between ENE and OS of penile cancer patients, we found great heterogeneity, with an *I*^2^ value of 74 %. We tried to find the origin of this heterogeneity by carefully reading all the 10 eligible studies. Even though the utilized protocols for lymph node dissection in penile cancer were variable, the basic protocol was similar in most of the included studies. However, in the study by Pandey et al. [14], the indication for lymph node dissection was extremely different from the others, and may be the source of heterogeneity. Their study used fine needle aspiration cytology (FNAC) to identify potential positive lymph nodes. Lymph node dissection was only conducted on FNAC positive inguinal lymph nodes or on those with clinically obvious or suspiciously enlarged nodes after the primary penile cancer surgery. As we know, the accuracy of FNAC in diagnosing LNM is limited, having a low specificity [8, 30]. Hence, the current EAU guideline does not recommended FNAC as a method for staging [19]. In the study by Pandey et al. (14), some patients with a positive inguinal lymph node missed synchronous lymph node dissection and only received salvage lymph node dissection after the inguinal lymph nodes became larger. This explains the high percentage of ENE positive and PLNM patients in their study [14].

Our study has some limitations. (1) The number of included studies was small; the analysis of CSS and PLNM each had only four eligible studies, and the OS analysis included only two studies. As a consequence, this limitation should be taken into consideration when interpreting the results. A well-designed study with a larger sample size will be needed to validate our results. (2) Great heterogeneity existed in the analysis for OS, but since only 2 studies were included, no subgroup analysis was performed to identify the reason for the heterogeneity. (3) We preferentially extracted HR from multivariable analysis, which was adjusted for other factors. However, the adjusted factors were not the same in HRs that were directly extracted from multivariate Cox analysis. We used the Kaplan-Meier curves to estimate the HRs in studies in that did not directly provide HRs. All of these factors, more or less, contributed to the observed heterogeneity. (4) We only searched the databases for studies in the English language, and ignored non-English or unpublished studies. (5) Adjuvant and/or neoadjuvant therapy might impact the prognosis of penile cancer [31, 32]. However, we failed to perform subgroup analysis on the percentage of patients that received adjuvant and/or neoadjuvant therapy due to the fact that not every included study provided this data.

## Conclusions

In summary, ENE is associated with worse CSS and OS for penile cancer patients. Our data also shows that patients with ENE in inguinal lymph nodes have higher risk of presenting with PLNM. However, because the number of included studies is small, well-designed studies with a large sample size are needed to confirm our results.

## Additional file

Additional file 1: **PRISMA 2009 Checklist.** (DOC 64 kb)

## Abbreviations

ENE: Extranodal extension
HR: Hazard ratio
OR: Odds ratio
CSS: Cancer-specific survival
CI: Confidence interval
PLNM: Pelvic lymph node metastasis

## References

[1] Backes DM, Kurman RJ, Pimenta JM, Smith JS (2009). Systematic review of human papillomavirus prevalence in invasive penile cancer. Cancer Causes Control.

[2] Chaux A, Netto GJ, Rodriguez IM, Barreto JE, Oertell J, Ocampos S (2013). Epidemiologic profile, sexual history, pathologic features, and human papillomavirus status of 103 patients with penile carcinoma. World J Urol.

[3] Parkin DM, Whelan SL, Ferlay J, Teppo L, Thomas DB (2002). Cancer incidence in five continents.

[4] Misra S, Chaturvedi A, Misra NC (2004). Penile carcinoma: a challenge for the developing world. Lancet Oncol.

[5] Jin F, Devesa SS, Chow WH, Zheng W, Ji BT, Fraumeni JF (1999). Cancer incidence trends in urban shanghai, 1972-1994: an update. Int J Cancer.

[6] Srinivas V, Morse MJ, Herr HW, Sogani PC, Whitmore WF (1987). Penile cancer: relation of extent of nodal metastasis to survival. J Urol.

[7] Ravi R (1993). Correlation between the extent of nodal involvement and survival following groin dissection for carcinoma of the penis. Br J Urol.

[8] Protzel C, Alcaraz A, Horenblas S, Pizzocaro G, Zlotta A, Hakenberg OW (2009). Lymphadenectomy in the surgical management of penile cancer. Eur Urol.

[9] Edge S, Byrd DR, Compton CC, Fritz AG, Greene FL, Trotti A (2010). American Joint Committee on Cancer, American Cancer Society. AJCC Cancer Staging Manual, 7th ed, 2010.

[10] Tobias-Machado M, Tavares A, Ornellas AA, Molina WR, Juliano RV, Wroclawski ER (2007). Video endoscopic inguinal lymphadenectomy: a new minimally invasive procedure for radical management of inguinal nodes in patients with penile squamous cell carcinoma. J Urol.

[11] Ficarra V, Akduman B, Bouchot O, Palou J, Tobias-Machado M (2010). Prognostic factors in penile cancer. Urology.

[12] Novara G, Galfano A, De Marco V, Artibani W, Ficarra V (2007). Prognostic factors in squamous cell carcinoma of the penis. Nat Clin Pract Urol.

[13] Djajadiningrat RS, Graafland NM, van Werkhoven E, Meinhardt W, Bex A, van der Poel HG (2014). Contemporary management of regional nodes in penile cancer-improvement of survival?. J Urol.

[14] Pandey D, Mahajan V, Kannan RR (2006). Prognostic factors in node-positive carcinoma of the penis. J Surg Oncol.

[15] Sun M, Djajadiningrat RS, Alnajjar HM, Trinh QD, Graafland NM, Watkin N, et al. Development and external validation of a prognostic tool for prediction of cancer-specific mortality after complete loco-regional pathological staging for squamous cell carcinoma of the penis. BJU Int. 2015;116(5):734-743. 10.1111/bju.12677 24552303

[16] Liu JY, Li YH, Zhang ZL, Yao K, Ye YL, Xie D (2013). The risk factors for the presence of pelvic lymph node metastasis in penile squamous cell carcinoma patients with inguinal lymph node dissection. World J Urol.

[17] Svatek RS, Munsell M, Kincaid JM, Hegarty P, Slaton JW, Busby JE (2009). Association between lymph node density and disease specific survival in patients with penile cancer. J Urol.

[18] Lughezzani G, Catanzaro M, Torelli T, Piva L, Biasoni D, Stagni S (2013). The Relationship Between Lymph Node Ratio and Cancer-Specific Survival in a Contemporary Series of Patients with Penile Cancer and Lymph Node Metastases. BJU Int.

[19] Hakenberg OW, Compérat EM, Minhas S, Necchi A, Protzel C, Watkin N (2015). EAU guidelines on penile cancer: 2014 update. Eur Urol.

[20] Lont AP, Kroon BK, Gallee MP, van Tinteren H, Moonen LM, Horenblas S (2007). Pelvic lymph node dissection for penile carcinoma: extent of inguinal lymph node involvement as an indicator for pelvic lymph node involvement and survival. J Urol.

[21] Zhu Y, Zhang SL, Ye DW, Yao XD, Jiang ZX, Zhou XY (2008). Predicting pelvic lymph node metastases in penile cancer patients: a comparison of computed tomography, Cloquet’s node, and disease burden of inguinal lymph nodes. Onkologie.

[22] Moher D, Liberati A, Tetzlaff J, Altman DG (2009). Preferred reporting items for systematic reviews and meta-analyses: the PRISMA statement. BMJ.

[23] Wells GA, Shea B, O’Connell D, Peterson J, Welch V, Losos M, Tugwell P (2003). The Newcastle–Ottawa Scale (NOS) for assessing the quality of nonrandomised studies in meta-analyses.

[24] Tierney JF, Stewart LA, Ghersi D, Burdett S, Sydes MR (2007). Practical methods for incorporating summary time-to-event data into meta-analysis. Trials.

[25] Egger M, Davey Smith G, Schneider M, Minder C (1997). Bias in meta-analysis detected by a simple, graphical test. BMJ.

[26] Begg CB, Mazumdar M (1994). Operating characteristics of a rank correlation test for publication bias. Biometrics.

[27] Zhu Y, Ye DW, Yao XD, Zhang SL, Dai B, Zhang HL (2011). New N staging system of penile cancer provides a better reflection of prognosis. J Urol.

[28] Lughezzani G, Catanzaro M, Torelli T, Piva L, Biasoni D, Stagni S (2014). The relationship between characteristics of inguinal lymph nodes and pelvic lymph node involvement in penile squamous cell carcinoma: a single institution experience. J Urol.

[29] Graafland NM, van Boven HH, van Werkhoven E, Moonen LM, Horenblas S (2010). Prognostic significance of extranodal extension in patients with pathological node positive penile carcinoma. J Urol.

[30] Ornellas AA, Kinchin EW, Nobrega BL, Wisnescky A, Koifman N, Quirino R (2008). Surgical treatment of invasive squamous cell carcinoma of the penis: Brazilian National Cancer Institute long-term experience. J Surg Oncol.

[31] Sonpavde G, Pagliaro LC, Buonerba C, Dorff TB, Lee RJ, Di Lorenzo G (2013). Penile cancer: current therapy and future directions. Ann Oncol.

[32] Pizzocaro G, Piva L (1988). Adjuvant and neoadjuvant vincristine, bleomycin, and methotrexate for inguinal metastases from squamous cell carcinoma of the penis. Acta Oncol.

